# InnB, a Novel Type III Effector of *Bradyrhizobium elkanii* USDA61, Controls Symbiosis With *Vigna* Species

**DOI:** 10.3389/fmicb.2018.03155

**Published:** 2018-12-18

**Authors:** Hien P. Nguyen, Safirah T. N. Ratu, Michiko Yasuda, Michael Göttfert, Shin Okazaki

**Affiliations:** ^1^United Graduate School of Agricultural Science, Tokyo University of Agriculture and Technology, Tokyo, Japan; ^2^Graduate School of Agriculture, Tokyo University of Agriculture and Technology, Tokyo, Japan; ^3^Institute of Genetics, Technische Universität Dresden, Dresden, Germany

**Keywords:** symbiosis, type III secretion system, effector, *Bradyrhizobium elkanii*, *Vigna* species

## Abstract

*Bradyrhizobium elkanii* USDA61 is incompatible with mung bean (*Vigna radiata* cv. KPS1) and soybean (*Glycine max* cv. BARC2) and unable to nodulate either plant. This incompatibility is due to the presence of a functional type III secretion system (T3SS) that translocates effector protein into host cells. We previously identified five genes in *B. elkanii* that are responsible for its incompatibility with KPS1 plants. Among them, a novel gene designated as *innB* exhibited some characteristics associated with the T3SS and was found to be responsible for the restriction of nodulation on KPS1. In the present study, we further characterized *innB* by analysis of gene expression, protein secretion, and symbiotic phenotypes. The *innB* gene was found to encode a hypothetical protein that is highly conserved among T3SS-harboring rhizobia. Similar to other rhizobial T3SS-associated genes, the expression of *innB* was dependent on plant flavonoids and a transcriptional regulator TtsI. The InnB protein was secreted via the T3SS and was not essential for secretion of other nodulation outer proteins. In addition, T3SS-dependent translocation of InnB into nodule cells was confirmed by an adenylate cyclase assay. According to inoculation tests using several *Vigna* species, InnB promoted nodulation of at least one *V. mungo* cultivar. These results indicate that *innB* encodes a novel type III effector controlling symbiosis with *Vigna* species.

## Introduction

Symbiotic relationships between legumes and soil bacteria, collectively called rhizobia, substantially contribute to agricultural production and the nitrogen cycle on the Earth (Broughton et al., 2000; Perret et al., 2000). Rhizobia induce the formation of specialized organs, known as root nodules, and are accommodated within them. The rhizobia in these nodules terminally differentiate into bacteroids, which are capable of reducing atmospheric nitrogen to ammonium assimilated by plants. In exchange, the host plants supply carbon sources and provide an appropriate environment for rhizobia (Oldroyd et al., 2011).

The nodulation process involves a complex exchange of molecular signals between rhizobia and plants that enables the hosts to distinguish compatible rhizobia from potential pathogens. Certain flavonoids exuded by host legume roots interact specifically with the rhizobial protein NodD, which binds to specific promoter sequences (called *nod* boxes) and activates the transcription of *nodulation* (*nod*) genes (Cullimore et al., 2001; Radutoiu et al., 2007). The products of *nod* genes synthesize rhizobial signal molecules called nodulation factors (NFs). NFs are recognized by host receptors (NF receptors, NFRs) that activate the host-signaling pathway, resulting in rhizobial infection and nodule organogenesis (Cullimore et al., 2001; Radutoiu et al., 2007; Madsen et al., 2010; Miwa and Okazaki, 2017).

Flavonoids from host legumes also induce secretion of specific proteins via the bacterial type III protein secretion system (T3SS). The T3SS was originally identified as a translocation system of effector proteins that function as virulence or avirulence proteins (Hueck, 1998). A similar system has been identified in numerous rhizobia (Viprey et al., 1998; Göttfert et al., 2001; Krause et al., 2002; Krishnan et al., 2003; López-Baena et al., 2008; Okazaki et al., 2009, 2010; Sánchez et al., 2009; Pérez-Montaño et al., 2016). The gene cluster for the rhizobial T3SS (*tts*) consists of genes encoding a secretion apparatus, secreted proteins and a transcriptional activator TtsI that induces the expression of *tts* genes by binding to conserved *cis-*elements termed *tts* boxes. Rhizobial type III secreted proteins are designated as nodulation outer proteins (Nops) (Krause et al., 2002; Marie et al., 2004; López-Baena et al., 2008; Wassem et al., 2008; Okazaki et al., 2009). Rhizobial T3SSs and secreted effectors, called T3 effectors, are involved in host-range determination and nodulation efficiency (Marie et al., 2003; Deakin and Broughton, 2009). Depending on the host plant, T3 effectors can have a positive, negative or neutral effect on symbiosis (Staehelin and Krishnan, 2015; Miwa and Okazaki, 2017).

*Bradyrhizobium elkanii* establishes symbiosis with a wide range of legumes, including soybean (*Glycine max*), mung bean (*Vigna radiata*), groundnut (*Arachis hypogaea*) and *Aeschynomene* spp. We previously reported that the T3SS of *B. elkanii* promotes nodule formation on soybean cultivar Enrei and *Aeschynomene* spp. but restricts nodulation on soybean cultivars carrying the *Rj4* allele (Faruque et al., 2015) and *V. radiata* cv. KPS1 (Okazaki et al., 2009). We identified five genes of USDA61 responsible for the incompatibility with *V. radiata* cv. KPS1 (Nguyen et al., 2017), one of which, designated as *innB* (***in****compatible* ***n****odulation B*), is preceded by a *tts* box and thus predicted to possess a T3SS-related function. Inoculation assays showed that *innB* is specifically responsible for the incompatibility with KPS1 but not for *Rj4* soybeans (Nguyen et al., 2017). In the present study, we further characterized the *innB* gene by transcriptional and protein analyses. We also observed infection properties and evaluated its symbiotic roles by inoculating several *Vigna* species. Our results reveal that *innB* encodes a novel type III effector that controls symbiosis with *Vigna* species.

## Materials and Methods

### Microbiological and Molecular Techniques

The bacterial strains used in this study are listed in Supplementary Table S1. *B. elkanii* USDA61 and mutant strains were grown at 28°C on arabinose–gluconate (AG) medium (Sadowsky et al., 1987) or peptone salts yeast extract (PSY) medium (Regensburger and Hennecke, 1983) and *Escherichia coli* strains were grown at 37°C on LB medium (Green and Sambrook, 2012). Antibiotics were added to the media at the following concentrations: for *B.* *elkanii*, polymyxin at 50 μg ml^-1^, kanamycin and streptomycin at 200 μg ml^-1^; for *E. coli*, kanamycin and streptomycin at 50 μg ml^-1^, tetracycline at 10 μg ml^-1^.

For the construction of InnB-3xFLAG translational fusion, the oligonucleotides 3FLAPstI and 3FLASphI (Supplementary Table S2) were annealed and cloned into the *Pst*I-*Sph*I site of the plasmid pK18mob (Schäfer et al., 1994), generating the plasmid pK18mob3xFLAG. The partial *innB* fragment was amplified by PCR using primers innB-PstI-fullF and innB-BamHI-fullR and then cloned into pK18mob3xFLAG to generate pInnB7593xFLAG. For the construction of InnB-Cya fusions, the streptomycin-resistance gene (*aadA*) was amplified by PCR using primers aadAfor and aadArev and then cloned into the DraI sites of the pSLC5 plasmid (Wenzel et al., 2010), generating pSLC5Sm. The *innB* fragment was amplified by PCR using primers innB-PstI-fullF and innB-BamHI-fullR and cloned into the *EcoR*I and *Xba*I sites of pSLC5Sm, generating pInnB759Cya. For complementation, *innB* and its promoteor region (Accession No. KX499541, Supplementary Data S1) was amplified by PCR using primers BeinnBSacI-InfuF and BeinnBKpnI-InfuR and cloned into the *Sac*I and *Kpn*I sites of the plasmid pBjGroEL4::DsRed2, generating pBjGroEL4::proinnB. The resulting plasmid was mobilized into the *innB*-deficient mutant BE53 (Nguyen et al., 2017) by conjugation (Krause et al., 2002) using the helper plasmid pRK2013. Integration of the plasmids into the chromosome of *B. elkanii* strains was confirmed by antibiotic resistance, PCR and sequencing. DsRed- and GusA-tagged *B. elkanii* strains were obtained by integration of the plasmids pBjGroEL4::DsRed2 (Hayashi et al., 2014) and pCAM120 (Wilson et al., 1995), respectively.

### Plant Assays

Seeds of mung bean (*V. radiata* cv. KPS1), black gram [*Vigna mungo* (L.) Hepper cv. PI173934] and soybean (*G. max* cv. Enrei) were surface sterilized and germinated as described previously (Nguyen et al., 2017). One day after transplantation, seedlings were inoculated with *B. elkanii* strains (1 ml of 10^7^ cells ml^-1^ per seedling). The plants were grown in a plant growth cabinet (LPH-410SP; NK Systems, Co. Ltd., Osaka, Japan) at 25°C and 70% humidity under a day/night regimen of 16/8 h. The symbiotic phenotypes including nodule number, nodule fresh weight and whole plant fresh weight were examined 8, 15, 30, or 35 days post-inoculation depending on the experiment.

### Microscopy

For microscopic analysis, nodules were fixed with 0.1 M cacodylate buffer containing 2.5% (vol/vol) glutaraldehyde in phosphate saline buffer at 4°C overnight. The fixed nodules were embedded in 5% agar and sectioned using a microtome (VT1000s; Leica Biosystems, Germany) and then observed under the microscope (SZX9; Olympus, Japan). GUS staining was performed as described previously (Okazaki et al., 2007).

### RNA Extraction and Real-Time RT-PCR

RNAs were extracted from *B. elkanii* cells as described by Babst et al. (1996). Briefly, *B. elkanii* strains were grown at 28°C on PSY medium on a rotary shaker at 180 rpm. When OD_600_ values of the culture reached 0.4, 10 μM of genistein was added and the cultures were sampled at 4 and 24 h, respectively. cDNA synthesis and real-time RT-PCR were performed as described by Yasuda et al. (2016). The RNA isolations and real-time RT-PCR analyses were performed at least twice in triplicate. Transcript levels were normalized to the expression of the housekeeping gene *atpD*, measured in the same samples (Wen et al., 2016).

### Purification and Analysis of Extracellular Proteins

For isolation of extracellular proteins, AG medium was inoculated with 1 : 100 dilution from a *B. elkanii* preculture and incubated at 28°C for 48 h. Supernatants were recovered from 500 ml of the bacterial cultures by two rounds of centrifugation (4000 ×*g* at 4°C for 1 h and 8000 ×*g* at 4°C for 30 min). The supernatants were then lyophilized, and extracellular proteins were extracted as described previously (Okazaki et al., 2009, 2010). Extracellular proteins were separated by 15% SDS-PAGE and stained with Coomassie brilliant blue (CBB) as described previously (Okazaki et al., 2010). For western blot analysis, protein samples were separated on a 4–15 or 4–20% SDS polyacrylamide gel, and identification of the target proteins was carried out as described by Hempel et al. (2009) using an antibody raised against FLAG and NopA. Briefly, the protein samples were transferred to polyvinylidene difluoride (PVDF) membranes (Bio-Rad, United States) and blocked with Western Blot Blocking Buffer (Fish Gelatin) (Takara, Japan). The immunoreaction was detected using an ECL Prime Kit (GE Healthcare, United Kingdom) and a LAS-3000 Luminescent Image Analyzer (Fujifilm, Japan).

### Adenylate Cyclase (Cya) Assay

To quantify cAMP levels, root nodules were collected at 18 dpi, immediately frozen in liquid nitrogen and ground to a fine powder. The nodule powder was suspended in a 5× volume of 0.1 M HCl (per nodule weight) and centrifuged. The supernatant was diluted to quantify cAMP concentrations in the detection range and subjected directly to cAMP measurement using a cyclic AMP (direct) enzyme immunoassay (EIA) kit (Cayman Chemical Company, Ann Arbor, MI, United States) according to the manufacturer’s instructions.

### Bioinformatic and Statistical Analysis

Amino acid sequences were aligned using MUSCLE or CLUSTAL W algorithms. A phylogenetic tree was constructed by the neighbor-joining method in MEGA 7.0 (Kumar et al., 2016). For statistical analysis, the data were subjected to analysis of variance (ANOVA), and a *post hoc* test (Fisher’s test at *p* ≤ 0.05) was done using Minitab statistical software version 16.0.

## Results

### InnB Is Exclusively Conserved Among Rhizobia

We previously demonstrated that *innB* of *B. elkanii* USDA61 was responsible for host-specific nodulation restriction on *V. radiata* cv. KPS1 (Nguyen et al., 2017). The presence of a conserved *tts* box motif in the promoter region of *innB* suggested its T3SS-related function (Supplementary Data S1). In addition, the N-terminal region of InnB exhibits characteristics of T3-secreted proteins: as detailed by Guttman et al. (2002) and Petnicki-Ocwieja et al. (2002), a high percentage of serines and the aliphatic amino acids proline and valine as the third or fourth residues (Supplementary Figure S1).

**FIGURE 1:**
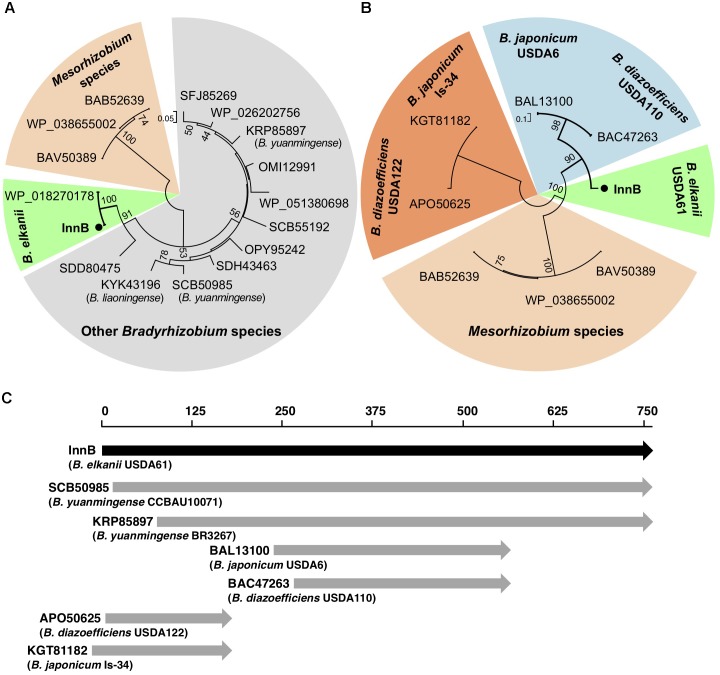
Phylogenetic trees and comparative alignment of InnB and its homologs. **(A,B)** Phylogenetic trees of homologs having a query coverage of at least 80% **(A)** and less than 80% **(B)**. **(C)** Alignment of InnB and its homologs in several rhizobial strains.

A BLASTP search revealed that homologs of InnB are present in T3SS-harboring rhizobia, but not in T3SS-harboring pathogens (Figure 1). Because the InnB homologs were highly diverse in length, we divided the selected homologs into high- and low-query-coverage groups and analyzed their phylogenetic relationships separately. Phylogenetic analysis of the high-query-coverage homologs uncovered three clearly distinct branches. InnB and its homologs in *B. elkanii* USDA76 (WP_018270178, 100% identity) and *Bradyrhizobium* sp. R5 (SDD80475, 74% identity) were separate from the two groups comprising *Mesorhizobium* and other *Bradyrhizobium* strains (Figure 1A). We also discovered three *Mesorhizobium* species containing InnB homologs, but their similarity to InnB was low. InnB homologs with a low query coverage were split into four different groups: *Mesorhizobium* strains, *B. elkanii* USDA61, *B.* *diazoefficiens* USDA110 and *B. japonicum* USDA6, *B. diazoefficiens* USDA122 and *B. japonicum* Is-34 (Figure 1B).

An amino acid alignment was conducted using selected homologs, namely, those in *B. yuanmingense* CCBAU10071 (SCB50985, 72% identity), *B. yuanmingense* BR3267 (KRP85897, 73% identity, not annotated), *B. japonicum* USDA6 (BAL13100, 71% identity), *B. diazoefficiens* USDA110 (BAC47263, 70% identity), *B. diazoefficiens* USDA122 (APO50625, 70% identity) and *B. japonicum* Is-34 (KGT81182, 70% identity) (Figure 1C and Supplementary Figure S1). The homologs in two strains, *B. yuanmingense* CCBAU10071 (SCB50985, query coverage 98%) and BR3267 (KRP85897, query coverage 89%), had many highly conserved amino acids that could be aligned with the InnB amino acid sequence. In contrast, the homologs in *B. diazoefficiens* and *B. japonicum* strains with a query coverage lower than 80% aligned with different parts of InnB (Figure 1C and Supplementary Figure S1). Interestingly, the homologs BAL13100 (in USDA6) and BAC47263 (in USDA110) were highly similar to the internal part of InnB, while those in USDA122 (APO50625) and Is-34 (KGT81182) aligned well with the N-terminus of InnB. All four of these homologs lacked amino acids that aligned with the amino acid residues 569 to 759 of InnB (Supplementary Figure S1).

### Expression of *innB* Is Dependent on Genistein and the Transcriptional Regulator TtsI

**FIGURE 2:**
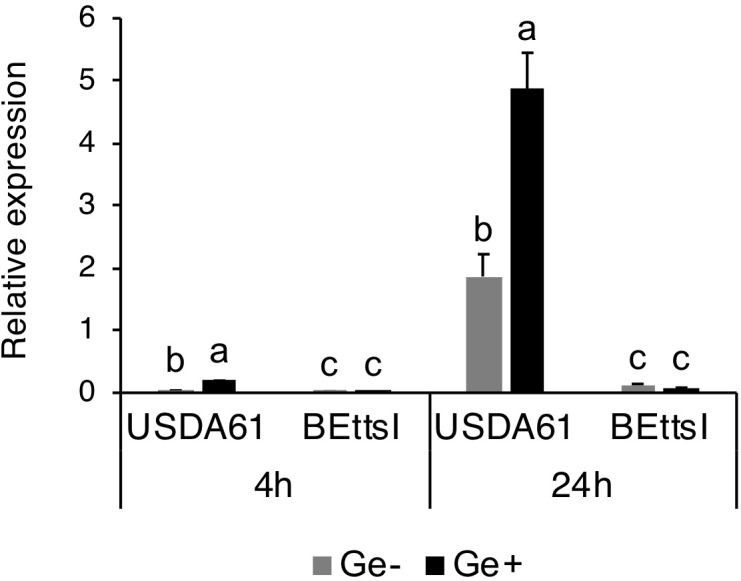
Expression of *innB* in wild-type and mutant *Bradyrhizobium elkanii* strains. Real-time RT-PCR was performed using total RNAs isolated from USDA61 and the *ttsI*-deficient mutant BEttsI grown in the absence (Ge–) or presence (Ge+) of the inducer flavonoid genistein (10 μM) after 4 and 24 h. The expression level of each gene was normalized relative to the *atpD* gene (ATP synthase) using the ΔΔCt method. The expression data are the mean of triplicates, and the error bars indicate standard deviations. Statistical analysis by Fisher’s method was performed to compare the relative expression levels of *innB* in USDA61 and BEttsI in the absence/presence of genistein at each timepoint, respectively. Means followed by different letters at the same timepoint are significantly different at the 5% level.

The presence of a conserved *tts* box motif in the promoter region of *innB* suggests that its expression is controlled by TtsI and host-derived flavonoids. We therefore analyzed the expression of *innB* in the wild-type USDA61 and the *ttsI*-deficient mutant BEttsI (Okazaki et al., 2013) in the presence and absence of genistein by real-time RT-PCR. Transcriptional levels of *innB*, *nopA*, and *nodC* genes in the wild-type background were increased upon induction with genistein (Figure 2). The *innB* and *nopA* showed a very similar expression and induction pattern with significantly higher expression after 24 h (Figure 2 and Supplementary Figure S2). In BEttsI, the expression of *innB* was completely abolished along with that of *nopA*, which suggests that *innB* expression was controlled by genistein and TtsI. Intriguingly, the expression of *nodC* was slightly reduced in the *ttsI* mutant background. However, it is unknown how TtsI might affect *nodC* expression.

### InnB Is Not Essential for the Secretion of Other Nops

**FIGURE 3:**
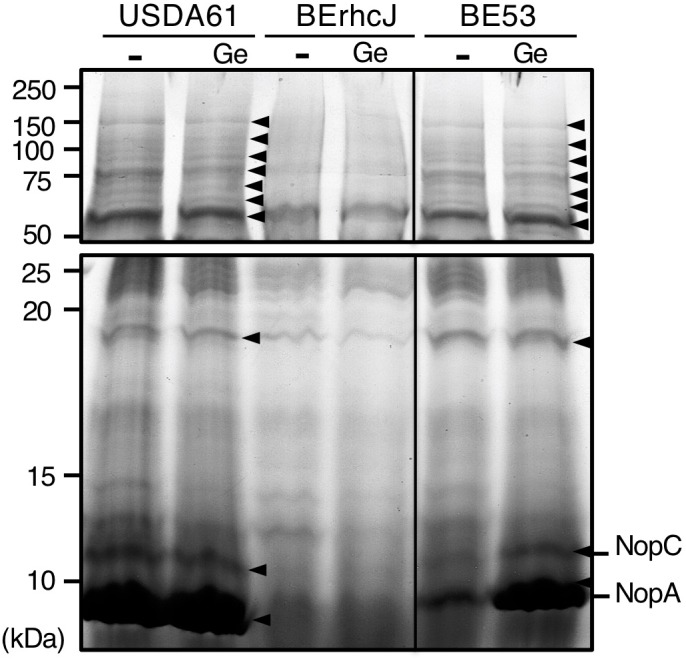
Extracellular protein profiles of *B. elkanii* strains. USDA61, BErhcJ, and *innB*-deficient mutant BE53 were cultured in the absence (–) or presence (Ge) of 10 μM genistein. The extracellular proteins were stained with Coomassie brilliant blue (CBB). Proteins were separated by 15% SDS-PAGE. Size-marker molecular masses (kDa) are shown on the left. Arrowheads indicate protein bands that were visible in cultures of USDA61 and BE53 but not BErhcJ.

To confirm that InnB is a T3 effector and that mutation of *innB* does not alter secretion of other Nops, we analyzed the extracellular protein profiles of strains USDA61 and BErhcJ, a mutant defective for T3 protein secretion (Okazaki et al., 2009), and the *innB*-deficient mutant BE53 in the absence or presence of genistein (10 μM). The proteins from bacterial culture supernatants were extracted and separated by SDS-PAGE (Figure 3). The protein profile of the BErhcJ mutant differed from that of USDA61, with several predicted T3SS-secreted proteins, such as NopA and NopC being absent. In contrast, the protein pattern of BE53 was similar to that of the wild-type USDA61 and secretions of NopA and NopC were detected. These results suggest that the mutation in *innB* was not essential for the secretion of other Nops. We also examined the secretion of InnB using the FLAG-tagged construct, however, we could not detect the FLAG-tagged InnB in culture supernatants of USDA61 (Supplementary Figure S3).

### InnB Is a T3 Effector Translocated Inside Plant Cells

**FIGURE 4:**
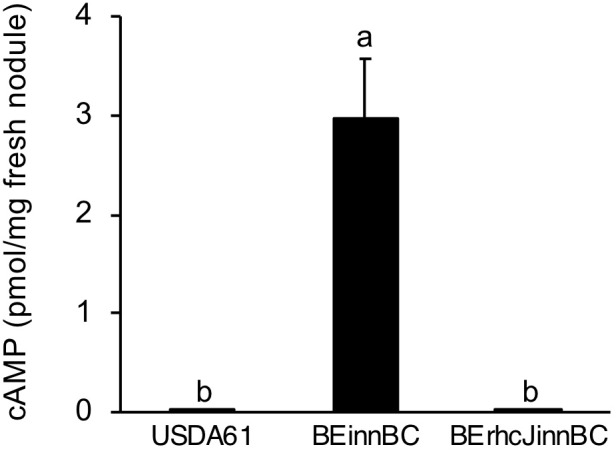
cAMP levels measured in nodules harvested at 18 dpi from the USDA61-compatible plant *Glycine max* cv. Enrei. Plants were inoculated with the rhizobial strains BEinnBC and BErhcJinnBC, which carry an *innB*-*cya* fusion in the USDA61 and BErhcJ background, respectively. The wild-type USDA61 contained no *cya* reporter. Nodules were randomly collected from at least four plants individually inoculated with each strain. The data are means of triplicates, and the error bars indicate standard deviations. Statistical analysis by Fisher’s method was performed to compare cAMP levels. Means followed by the different letters are significantly different at the 5% level.

To confirm whether InnB is a T3 effector that functions in host cells, its translocation was analyzed using adenylate cyclase (Cya) as a reporter. In the presence of ATP and a calmodulin-like protein, cAMP production is catalyzed by the Cya enzyme only within eukaryotic cells (Sory and Cornelis, 1994). Once the Cya-fused effector is delivered to the host cell, cAMP production will be detected. In this study, Cya fused to the carboxy terminus of InnB was integrated into the *B. elkanii* strains USDA61 and BErhcJ, resulting in the mutants BEinnBC and BErhcJinnBC, respectively. The translocation of InnB was confirmed using fresh nodules of the USDA61-compatible cultivar *G. max* cv. Enrei harvested at 18 dpi. A low level of cAMP was detected in nodules induced by the wild-type USDA61 and the BErhcJinnBC mutant expressing the InnB-Cya fusion (Figure 4). However, the cAMP level was significantly higher in nodules formed by USDA61 containing the InnB-Cya fusion, which suggests that InnB was translocated into the host cell.

### InnB Negatively Affects Nodulation of *V. radiata*

We previously reported that the insertion of a transposon in *innB* abolishes nodulation incompatibility between USDA61 and KPS1 (Nguyen et al., 2017). To confirm that the symbiotic phenotype was induced by inactivation of *innB* and not by an additional mutation, we complemented the BE53 mutant with the *innB* gene and its promoter region. The resulting complemented strain exhibited a symbiotic phenotype similar to wild-type USDA61 (Supplementary Figure S4). These results confirm that *innB* was responsible for the altered nodulation phenotype on KPS1.

**FIGURE 5:**
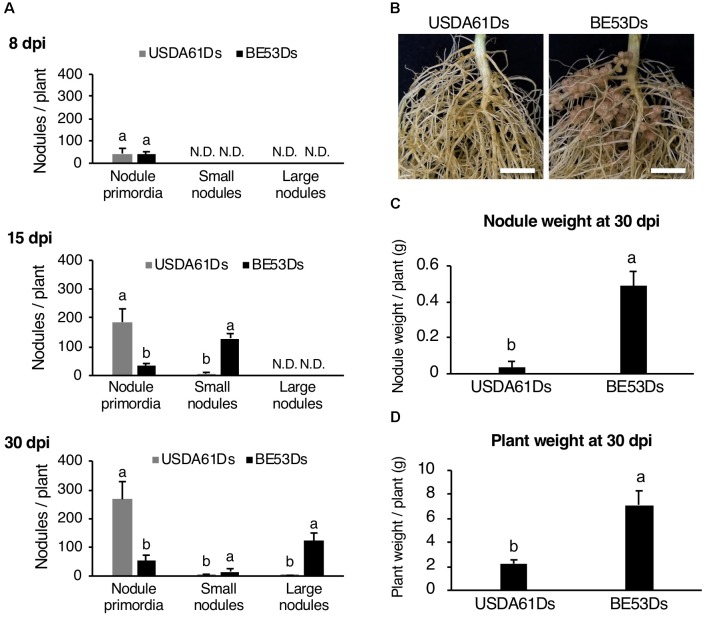
Nodulation properties of *Vigna radiata* cv. KPS1 inoculated with the *B. elkanii* USDA61Ds and BE53Ds. **(A)** The number of nodule primordia and small and large nodules at 8, 15, and 30 dpi. N.D., not detected. **(B)** Roots of KSP1 plants at 30 dpi; scale bars: 1 cm. **(C)** Nodule weight and **(D)** plant weight of KPS1 plants at 30 dpi. The data shown are the means of at least five plants, and the error bars indicate standard deviations. Statistical analysis by Fisher’s method was performed to compare the symbiotic phenotypes obtained with USDA61Ds and BE53Ds. Means followed by different letters are significantly different at the 5% level.

To further characterize the negative impact of InnB on nodulation, we monitored the nodulation process of KPS1 inoculated with DsRed-tagged *B. elkanii* strains. Early in nodulation (8 dpi) wild-type and mutant induced a similar number of nodule primordia (Figure 5A). However, at later stages (15 and 30 dpi), plants developed a higher number of nodule primordia if infected with the wild-type as compared to the infection with the *innB* mutant (5- to 20-fold). In contrast, almost no nodules were formed with the wild-type strain but the *innB* mutant was a very efficient nodulator (Figures 5A–C). In line with that, nodule weight and plant weight were significantly increased with BE53Ds-inoculated plants (Figure 5D). Similar results for the time point 40 dpi were observed previously (Nguyen et al., 2017).

### InnB Abolishes Infection and Nodule Organogenesis in *V. radiata*

**FIGURE 6:**
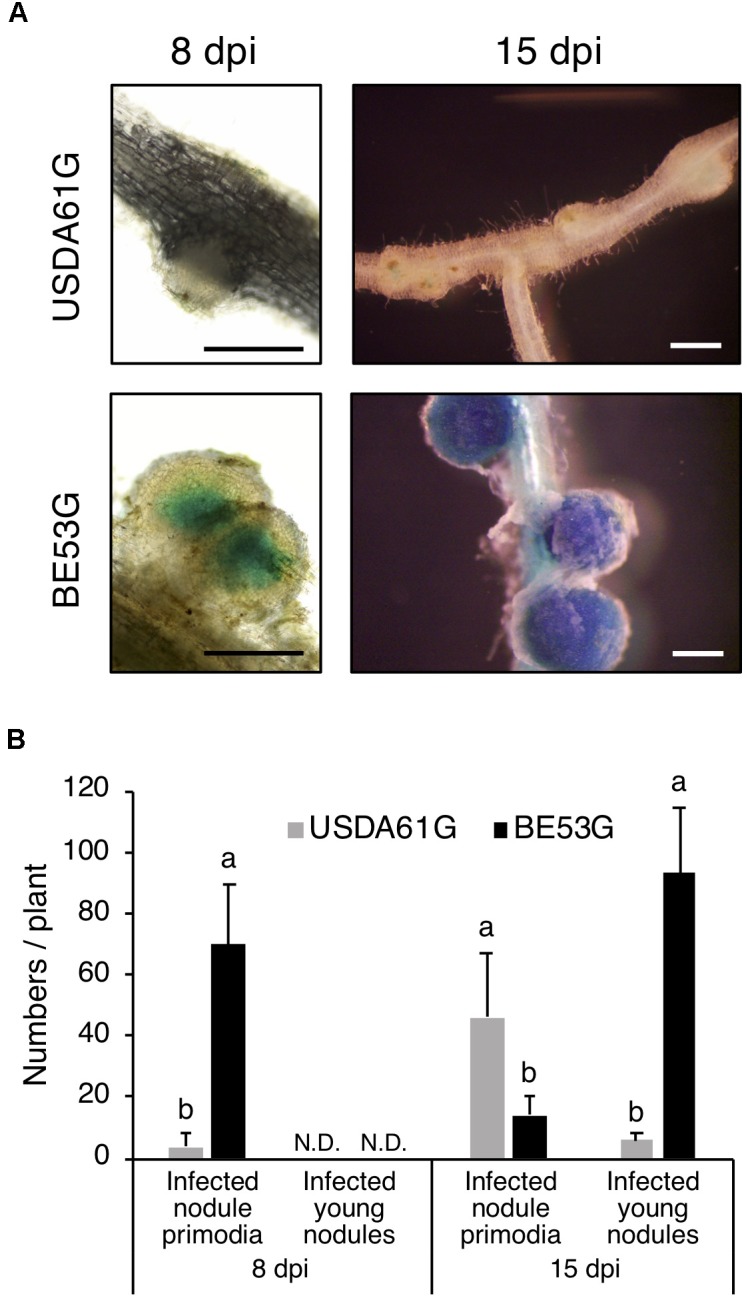
Infection properties of *V. radiata* cv. KPS1 inoculated with *Bradyrhizobium elkaii* USDA61G and BE53G. **(A)** Infected nodule primordia and small nodules. Scale bars: 500 μm. **(B)** The number of infected nodule primordia and small nodules formed on KPS1 plants. The data shown are means of five plants, and the error bars indicate standard deviations. Means followed by different letters at the same timepoint (dpi) are significantly different at the 5% level.

To explore the effect of *innB* on infective properties of *B. elkanii*, we investigated the infection process in KPS1 plants using GUS-tagged strains of USDA61 and BE53. Examination of early infection events in KPS1 roots revealed that USDA61 and BE53 both infected KPS1 root hairs and formed nodule primordia. However, the number of infected nodule primordia and small nodules was significantly different between USDA61 and BE53 (Figure 6). At 8 dpi, BE53 efficiently infected KPS1 roots, thereby resulting in a higher number of infected nodule primordia than those of the wild-type. Most nodule primordia in KPS1 plants inoculated with USDA61 were uninfected (Figure 6). In contrast to the infection phenotypes observed at 8 dpi, USDA61 induced a higher number of infected nodule primordia at 15 dpi than did BE53G. Many efficiently infected small nodules instead were formed on roots of KPS1 inoculated with BE53 (Figure 6). The small nodules formed by all plants inoculated with BE53 were deep blue, probably because of the high number of active rhizobia inside (Figure 6).

### InnB Promotes Nodulation of *V. mungo*

**FIGURE 7:**
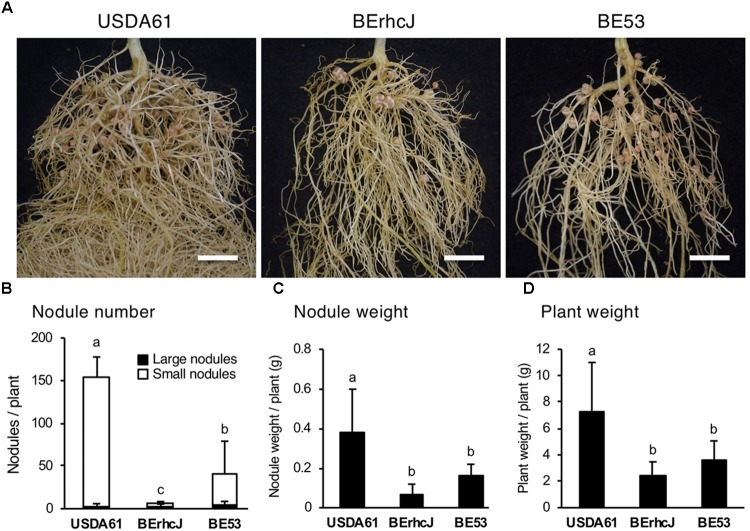
Symbiotic properties of *Vigna mungo* cv. PI173934 (black gram) inoculated with *B. elkanii* strains. **(A)** Roots of black gram inoculated with *B. elkanii* strains; scale bars: 1 cm. Nodule number **(B)**, fresh nodule weight **(C)**, and fresh plant weight **(D)** of *V. mungo* at 35 dpi. White and black bars in **(B)** represent the number of small (<2 mm) and large (≥2 mm) nodules, respectively. The data shown are the means of at least 10 plants from two independent assays. The error bars indicate standard deviations. Means followed by different letters are significantly different at the 5% level.

To explore the importance of InnB in symbiosis with legumes, we conducted inoculation tests using several *Vigna* species. Among these species, *V. mungo* (L.) Hepper cv. PI173934 formed numerous nodules following inoculation with USDA61 (approximately 140 nodules per plant) (Figure 7). In contrast, BErhcJ and BE53 induced significantly lower numbers of nodules, and plant weights were decreased. These observations indicate that the T3SS promotes symbiosis with *V. mungo* and that InnB is one of the positive effectors for the symbiosis. Intriguingly, BE53 induced a significantly higher number of small nodules than BErhcJ, which suggests that USDA61 harbors an additional positive effector.

## Discussion

We previously identified five novel *B. elkanii* genes that are responsible for the incompatibility with *V. radiata* KPS1. One of the genes, *innB*, encodes a hypothetical protein that contains a *tts* box in its promoter region and thus seems to possess T3SS-related functions (Nguyen et al., 2017). As previously reported, the *innB* mutant BE53 can nodulate KPS1 but not *Rj4* soybean (Nguyen et al., 2017), which indicates that InnB causes a highly specific incompatibility with KPS1 but not with *Rj4* plants. In the present study, we analyzed the *innB* gene by examining gene expression, protein secretion and symbiotic phenotypes.

Homologs of the InnB protein were found in T3SS-harboring rhizobia, but not in other plant-associated bacteria. Other rhizobial T3-secreted proteins, such as NopC, NopI, NopL and NopP, have also been identified as *Rhizobium*-specific and have no homologs in plant or animal pathogens (Deakin and Broughton, 2009; Jiménez-Guerrero et al., 2015). These reports imply that InnB has a symbiosis-specific function. Notably, homologs in *B. japonicum* (strains USDA6 and Is-34) and *B. diazoefficiens* (strains USDA110 and USDA122) are highly similar to either the N-terminal or internal region of InnB (Figure 1 and Supplementary Figure S1). Our preliminary experiment in agreement with Sugawara et al. (2018) showed that USDA110 was not incompatible with KPS1, thus suggesting that the homolog of USDA110 (BAC47263) lacks a functional domain for inducing incompatibility. In contrast, InnB homologs in other bradyrhizobia, such as *B. elkanii* strain USDA76 and *B. yuanmingense* strains CCBAU10071 and BR3267, showed a high degree of query coverage, suggesting that these proteins have common functions in symbiosis.

Similar to other *nop* genes, the transcription of *innB* was found to be regulated by flavonoids and the transcriptional regulator TtsI. In addition, the different transcriptional levels of *nodC* in the absence and presence of genistein in the wild-type and *ttsI* mutant suggest that *nodC* expression is slightly reduced in a TtsI mutant background (Figure 2 and Supplementary Figure S2). The mechanism underlying the reduced *nodC* expression remains unclear. Notably, the transcriptional levels of *nopA* and *nodC* are lower than that of *innB*, suggesting that they might function differently in protein secretion and nodulation process (Supplementary Figure S2). Although transcription of *tts* genes were clearly inducible by genistein, the addition of genistein did not significantly change the extracellular protein patterns of *B. elkanii* strains (Figure 3). These results suggest that the T3SS is activated without genistein under the tested conditions. Therefore, unidentified regulators or inducers are likely be involved in the regulation of *tts* genes in *B. elkanii*.

An analysis of extracellular proteins confirmed that InnB is not essential for the secretion of other proteins. It has been shown that the mutations of genes encoding T3SS components, such as *nopA* and *nopB*, significantly affect the secretion of other secreted proteins and nodule formation (Deakin et al., 2005). In our experiment, Nops, such as NopA, NopC and NopX, were visible in both BE53 and USD61 extracellular protein samples, but not in those of T3SS-deficient BErhcJ (Figure 3), thus indicating that InnB is a secreted protein and not a component of the secretion apparatus.

The InnB protein, which was predicted to have a molecular mass of approximately 83.11 kDa, was not clearly detected by SDS-PAGE (Figure 3). To further confirm the secretion of *innB*, we constructed a 3xFLAG-tagged *innB*. The generated construct was transferred to either USAD61 or a *ttsI* mutant, generating BEinnBF and BEttsIinnBF, respectively. However, we could not detect InnB fragment in the culture supernatant of both strains by western blotting (Supplementary Figure S3B). Notably, the InnB homolog in *B. diazoefficiens* USDA110 was also not detected in the extracellular proteins of USDA110 (Hempel et al., 2009). It is likely that the InnB protein was secreted at quite low levels or secreted InnB proteins undergo a certain modification or degradation.

The translocation of InnB into soybean nodule cells was verified by a Cya reporter assay (Figure 4). Translocation of rhizobial T3 effectors such as NGR234 NopP (Schechter et al., 2010), NopC (Jiménez-Guerrero et al., 2015), HH103 NopL (Jiménez-Guerrero et al., 2017), and USDA110 NopE (Wenzel et al., 2010) into host cells has been similarly confirmed by Cya assays. The InnB-induced cAMP accumulation observed in our study was much lower than that of other Nops. This result may reflect the lower secretion and translocation of InnB compared with other Nops.

In *V. radiata*, USDA61 induced numerous nodule primordia, but the presence of InnB halted the subsequent nodulation process. While BE53 induced a higher number of infected small nodules on KPS1 roots at 15 dpi, the number of nodule primordia formed by USDA61 increased continuously until 30 dpi (Figures 5, 6). The failure of small nodule formation correlates well with an increase in nodule primordia formation (Figure 5). Taken together, KPS1 halted USDA61 infection and nodule organogenesis before small nodule formation due to the presence of InnB. Probably, the InnB secreted into host cells interacts specifically with *Vigna* resistance (*R*) genes and triggers immune responses (effector-triggered immunity), thus resulting in host defense responses and consequently halting infection and nodulation by USDA61.

Although nodulation on KPS1 by USDA61 was heavily restricted, a few nodules were occasionally observed on the roots (Figure 5). According to previous research, USDA61 can infect host legumes such as *Arachis hypogaea* (Ibáñez and Fabra, 2011) and *Aeschynomene* spp. (Bonaldi et al., 2011) via “crack entry” (intercellular infection). In soybean, infection via crack entry to form nodules is mediated by the T3SS of *B. elkanii* and may induce a defense response in *Rj4* plants (Okazaki et al., 2013; Yasuda et al., 2016). However, T3SS-mediated crack entry infection is rare and weak and only results in the formation of several small nodules or bumps (Yasuda et al., 2016). In contrast, rhizobial infection through infection threads (ITs) can induce a stronger host defense that is completely able to block nodule formation (Yasuda et al., 2016). We observed that USDA61, but not the *innB*-deficient mutant, failed to induce root hair curling and only weakly infected KPS1 roots via ITs; nevertheless, a few large nodules occasionally formed at the base of lateral roots (Supplementary Figure S5). In addition, most USDA61-induced nodules were more poorly infected than those induced by BE53 (Supplementary Figure S5). These observations imply that the T3 effector-triggered host defense occasionally fails to completely block the nodule formation of USDA61-infected cells. In these cases, the USDA61 infection may occur via crack entry of the lateral roots of KPS1.

In *V. mungo*, BE53 induced less nodules than USDA61, indicating that the InnB plays a beneficial role in the symbiosis with this species. It was reported that the *V. mungo* plants were nodulated by *Bradyrhizobium* spp. (closely related with *B. yuanmingense*) but not by *B. japonicum* or *B. diazoefficiens* (Appunu et al., 2009). Notably, InnB homologs with a high coverage ratio were specifically found in *Bradyrhizobium* spp., but not in *B. japonicum* or *B. diazoefficiens* (Figure 1). These suggest that the symbionts of *V. mungo* specifically possess InnB for enhancing the interaction with this host plants. Intriguingly, the number of BE53-formed small nodules is higher than that formed by infection with BErhcJ, implying the presence of an additional positive effector. Further studies are required to elucidate the specific functions of the *Rhizobium*-specific T3 effector InnB and the additional effector in nodulation among different legumes.

In summary, we have presented evidence that the *innB* gene of *B. elkanii* encodes a novel T3 effector involved in the control of symbiosis with *Vigna* species. The conservation of InnB homologs among rhizobia suggests a symbiosis-specific function for *innB*, especially in interactions between bradyrhizobia and *Vigna* spp.

## Author Contributions

HN and SO designed the research and wrote the paper. HN performed the research and analyzed the data. SR, MY, and MG contributed new reagents and analytic tools.

## Conflict of Interest Statement

The authors declare that the research was conducted in the absence of any commercial or financial relationships that could be construed as a potential conflict of interest.
