# Supplementary material for: InnB, a Novel Type III Effector of Bradyrhizobium elkanii USDA61, Controls Symbiosis With Vigna Species
Source: Front Microbiol. 2018 Dec 18;9:3155. doi: 10.3389/fmicb.2018.03155 (PMC6305347; doi:10.3389/fmicb.2018.03155)
Supplement: Supplementary file 1 [file Data_Sheet_1.pdf]

Supplementary Material

InnB, a Novel Type III Effector of Bradyrhizobium elkanii USDA61, Controls Symbiosis With Vigna Species

Hien P. Nguyen, Safirah T. N. Ratu, Michiko Yasuda, Michael Göttfert and Shin Okazaki* *Correspondence: Shin Okazaki: sokazaki@cc.tuat.ac.jp

1 Supplementary Data

AGGAACGCTTACGGGCTGTCTTCTCATTTTTAAGCGGCTCTCGGGCCAATAGTGCGCACGAGAGCGTGCTT
GCGCTGGGGCGTCCCGTCGGCAGCAACTGAAATTGGGGCAGGGCACGGCCGTCGCACCTCGCCGGCTCCTT
GCTGCCCCTTCAGAAGAGATCAACGCCAACGGCATTGCCCGTTGTGAATTCTTGTTCTCGTCAGCTTTTCG
AAAGCTAGGTCCCATAAAAATCCAGAACCTGCCTTCGCTTCACGGCTGCCACTTAGCGGCCGTGCAAGCGT
GCGGATTGCGCAGGGCCTACACCATCACGAGGAGAGCAGG**ATGGATCCAGTCGACCCATTCTTCGATTCAG**
GAGCTTGGATGGAGGCGTACGCCGCCCGACAGGGGCGAGCGGGGCAGCCAGGGGGCAACGCAAGCCAGCAA
GGCGATTTTGAGCGGCGGATGGAGGATTTGCAGCTCGATTCGTCTGATGAGAGTTCCGCAAAGTCGAGTTC
GCCGGACCAGGCTCCCACAGGGGCCGGGCGAGCAGTTCGACGGGACGAGCTCGGCGGTTCGATGCGGGTGC
CGCCACGCTCGGATTTTAGGGACAGCGCACGGGGCGATGCGCGGCGGAGCTTAGACATTCCCCGCTTTCAG
CTTGATGCTCCGGCCCAAGTGCCGCAGTCCAGTTTGCGCGATGATCTATTCAGCAGCGCGCGCTACAGCTT
GCCGGACGCGGAGCCTGGGGTTCAGGCCAGGAGCGGTAAAAGCCGCGGACTGTGGTCGCGCTTCAAGTCGG
GGGTCGGCAAGGCCTTTGGAGGGAGTAGCAAGTCCTCGCGGGAGACCGCGCAGTCGGATGCTTCCTCGACG
AGTTTTCGCGTCGATTACGCCAGACAGCCGGGCCGGACGCGCGGGGTCGATCCGGAAGACGAGGCGCTCAT
CAAAGAGTTTAGACAAAGAGCAGTCCGCAACCGCACCGACGGCACCGCAGCCGGCAACCTCACCGACGGCA
CCATCAGAAATGCGGCAGCCGACCTGCGCATTTTGAGCGCGCGCCTGAATGACAACGATAGGCCCTCAATT
GCGGATCGCATTCGCCTCGAGATGGAAAATGCGCAACTGGAAGATCCGGAGGTGGAAAACCACCAGCTCGA
AGCTGAGCTCGACCAAGATGTCGACACCTATGCAGCGGACCGCGCAAGGCGTATCAAAGCTGCCTTGAAAA
AGCTCCGTGAGGTCGGCGCCGGAAACACCTTGTCAGCCGATCTCCGTCGCCTGGCTCCCCATTCCGCGGAC
GCGACCCTCATCGGCATGTGGGCCGCGGCGGAAAAGGCGACACGCAGGGTTGAGCCGAAGACGATCGACAG
GCAGGCTCGCCGAATGTCGAGGCTGAGTGAATGGCTGCAAAAGCACGATAAGCAGGCCATCGCCGGCCGGC
TCTTCACCTCCGGGCTCGACCAGGATGTCGCGGAGTATAGGCAAGAAACCGAAGACGGCAAAATCAAAGCT
GATTTGCTCAGGCTTGGCCGCTACCAGCAAATCCTTGAGGCGAACAGGGCCCTCGGCTTGCACCCTGCCGA
GGATGCGGGGCAGCCCGTCGCCGAAGGCGCCCGACAGGCGCACTCGCCGCAGGAGGTCCCGGCAACGCCAG
CCACGCCGAGTGCGGGAGCGTGGGATTGGCTTGGTGAGCAGATCCACGGACCCACCTCATCACTGGCAGTG
CCGCACCACGGTTGGCAGGCCAGCTCGTCGCAGCAGCTTCCCGCAACATCGGCCAGCCCGAGCGCCGGGGC
TTGGGATCGGTCCAGCGAGCAGATTCTCCAACCCGCCGCACCTGTGCAAGCGCCCTATTGGCTCCCGCAAC
CCAATCTGCCGCACGGGCTTCCTGCGACTCCAGCCAGCCTAAGCCAAGGGGCCTGGGATTGGCTCGGGCAA
CAGATGCAGGAACCCGCCTCACCATCGTCGGTGAGGCCTCGGTCATCAAACATCTACAGCGGTCTGGATCC
CTTGGTTGATTTGGATCCGTCCACACCGCACGACTTGCACGACGATGCACGCTCTGCGCCCGCACCTGAAT
TTCGCAGAGCGCCGTCATTCGCTGGACCGTCCGGGGGCGCCCAGGAACTGCGGGATATTGGGGCCGTCGTT
GGCTCGGATTGGCGTCACGGCTCCCAAGCGGCCTCGGACGTGTTGGTTGACGTCCTCGGCAACATCAATCT
GCTGCCAAACCAGTTCGGGCCAAGTCAGTTTATGATCAACGGTGAGCGCTACTCGGCCACATTCGGACCAG
GAGGGCGCACGGATGTCCGCCTGATCCATCATCCGCGCCCGAGCTACCTTGATGAAGCTGGGCCATCCCAG
CCGCTTCATCATCCACCGCAGATCGTCCAAGCCGAGCAACCCCGTGAGGGCCTACGGACCTTGGGTATCT
GATCCGCGGCGGATGGGAGCACCGCGAGCGGTTCCTTCCGCCTTACCTTGTCCGCGTCCTGCAGGGGGAAC
GCATCATGCCGCAGGCTGGGCGTCCAACCTATTTCCAGATCCGCGGTGTGCCCTACAGGGGCGAGTTGGAT
GAAAGCGAGGGGCGCCAGCGCGTTCGCATCTATCCTGAACGTGGCTGA

Supplementary Data S1. The *innB* gene (accession number KX499541) and its promoter sequence of *Bradyrhizobium elkanii* USDA61. The preceded conserved *tts* box motif is shown in the square box, and the *innB* sequence is shown in bold.

2 Supplementary Figures and Tables

2.1 Supplementary Figures

```
           1                                                     50
     InnB  .......... ..MDPVDPFF DSGAWMEAYA ARQGRAGQPG GNASQQGDFE
 SCB50985  .......... .......... .....MREYA ALQERTAQRG DNQRERRGFE
 KRP85897  .......... .......... .......... .......... ..........
 BAL13100  .......... .......... .......... .......... ..........
 BAC47263  .......... .......... .......... .......... ..........
 APO50625  .......... ..MDPIDPFF DSGAWMREYA ALPGRAGQRR GDSSEQGDFE
 KGT81182  MLQGLYKHEE SRMDPVDPFF DSGAWMREYA ALPGRAGQRR GDSSEQGDFE

           51                                                   100
     InnB  RRMEDLQLDS SDESSAKSSS PDQAPTGAGR AVRRDELGGS MRVPPRSDFR
 SCB50985  RRLEDLSLDP SDESN..SNS PDRAPAGGGR AVRPEDFGGS MRVPPDSAFR
 KRP85897  .......... .......... .......... .......... MRVPPHSAFG
 BAL13100  .......... .......... .......... .......... ..........
 BAC47263  .......... .......... .......... .......... ..........
 APO50625  KRMEDLHLDP SDQSK..STS PDRPPAGGGR AVRRDDFGGS MRAPPHSTFR
 KGT81182  KRMEDLHLDP SDQSK..STS PDRPPAGGGR AVRRDDFGGS MRAPPHSTFR

           101                                                  150
     InnB  DSARGDARRS LDIPRFQLDA PAQVPQSSLR DDLFSSARYS LPDAEPGVQA
 SCB50985  ESALGGTRRS VDIPSFQLPA SAQVPQSGLR DDPFSSARYS FPDAESAASA
 KRP85897  ESALDGARRS ADIPTFHLPA SAQ.PQSSLR DDLFSSARYS FPDAEPAAVA
 BAL13100  .......... .......... .......... .......... ..........
 BAC47263  .......... .......... .......... .......... ..........
 APO50625  ESALGDTRRS VDIPSFQLTA ATQVPQSSLR DDLFSSAR.. ..........
 KGT81182  ESALGDTRRS VDIPSFQLTA ATQVLQSSLR DDLFSSAR.. ..........

           151                                                  200
     InnB  RSGKSRGLWS RFKSGVGKAF GGSS..KSSR ETAQSDASST SFRVDYARQP
 SCB50985  KSGKSGGLWS RFKSGIGKAF GGSSSEKYSR NAGQGDAFST TLRIDYARQP
 KRP85897  KSGKSRGLWS RFKSGIGKAL GGSGSEKSPR EADQSDVFST NLRIDYARQP
 BAL13100  .......... .......... .......... .......... ..........
 BAC47263  .......... .......... .......... .......... ..........
 APO50625  .......... .......... .......... .......... ..........
 KGT81182  .......... .......... .......... .......... ..........

           201                                                  250
     InnB  GRTRGVDPED EALIKEFRQR AVRNRTDGTA AGNLTDGTIR NAAADLRILS
 SCB50985  GRTRAVPEED EVLIRDFRNK A......... AGNLTDGTIK NAAADLRHLS
 KRP85897  GRTRGVPAAD EELIEDFRNK A......... AGNLSDGTIK NAAADLRHLS
 BAL13100  .......... .......... .......... .......... ..........
 BAC47263  .......... .......... .......... .......... ..........
 APO50625  .......... .......... .......... .......... ..........
 KGT81182  .......... .......... .......... .......... ..........
```

```
            251                                                  300
    InnB    ARLNDNDRPS IADRIRLEME NAQLEDPEVE NHQLEAELDQ DVDTYA..AD
SCB50985    ARLSTAGRPS IADRIQRESE NAQQDNPEVE NRQLEAELDE DVDTYA..KD
KRP85897    ARLSDAGRPS IADRIRRELE NAQLENPDVE NHQLEAELDQ DVDAYTHTKD
BAL13100    .......... ........ME NAQQDNPEVE NHQLEAELDE EVDRYA..GR
BAC47263    .......... .......... ........ME NHQLEAELDE EVDRYA..GR
APO50625    .......... .......... .......... .......... ..........
KGT81182    .......... .......... .......... .......... ..........

            301                                                  350
    InnB    RARRIKAALK KLREVGAGNT LSADLRRLAP HSADATLIGM WAAAEKATRR
SCB50985    RSRRIKAALK KLREVGAGNA LAPDLRRLAP HRADAILIDM WSAAEKAAHR
KRP85897    RGRRIKAALK KLREVGAGNT LAADVRRLAP HAEDATLIRM WAAAEKATGR
BAL13100    RGTRIKAALK KLREVGAGNA LAPDLRRLAP HGADATLIHM WAAAEKATRR
BAC47263    RGTRIKAALK KLREVGAGNA LAPDLRRLAP HGADATLIHM WAAAEKATHR
APO50625    .......... .......... .......... .......... ..........
KGT81182    .......... .......... .......... .......... ..........

            351                                                  400
    InnB    VEPKTIDRQA RRMSRLSEWL QKHDKQAIAG RLFTSGLDQD VAEYRQETED
SCB50985    IEPETIDRQA RRMYRLSEWL QTHDRPAMAG RLSTPALDRD VEEYRRETKD
KRP85897    IDPKTIDRQA RRMFRLSEWL QTHDRLPIAG RLSTPAFLQD VEEYRRETDD
BAL13100    IEPKTIDRQA RRMSKLSEWL RTHDRPPMAG RLSTPAFVRD LEEYRRETND
BAC47263    IEPKTIDRQA RRMSKLSEWL RTHDRPPMAG RLSTPAFVRD LEEYRRETND
APO50625    .......... .......... .......... .......... ..........
KGT81182    .......... .......... .......... .......... ..........

            401                                                  450
    InnB    GKIKADLLRL GRYQQILEAN RALGLHPAED AGQPVAEGAR QAHSPQEVPA
SCB50985    GKIKPDLVKL GQYEQILEAN RALGLRPPDD PGQPSWEAAR QPHSMQEPPA
KRP85897    SKINPDLVKL RQYEQILEAN RALGFRTPED PGLPSREAAR QPHTLQEPPA
BAL13100    KKINADLVKL GGYEQILEAN RALGLRPPED PGQPSWEAAR QPHALQGPPA
BAC47263    KKINADLVKL GGYEQILEAN RALGLRPPED PGQPSWEAAR QPHALQGPPA
APO50625    .......... .......... .......... .......... ..........
KGT81182    .......... .......... .......... .......... ..........

            451                                                  500
    InnB    TPATPSAGAW DWLGEQIHGP TSSLAVPHHG WQASSSQQLP ATSASPSAGA
SCB50985    TPATPSAGAW DWLGEQIHGP TTSMRAPSD. WQAGSSQQLP AAPATPSAAA
KRP85897    TPATPSAGAW DWLGEQIHGP TTSMPTPPSG WQAGASQLLP ATPATPSTGA
BAL13100    TPASPSAGAW DWLGEQIHGP NISMPAPPSH WQAGSSQQLP ATPATPSAGA
BAC47263    TPASPSAGAW DWLGEQIHGP NISMPAPPSH WQAGSSQQLP ATPATPSAGA
APO50625    .......... .......... .......... .......... ..........
KGT81182    .......... .......... .......... .......... ..........

            501                                                  550
    InnB    WDRSSEQILQ PAAPVQAPYW LPQPNLPHGL PATPASLSQG AWDWLGQQMQ
SCB50985    RDWIGEQIHE PASPVPAPHW AAQAHPPLEL PATPATPSQG AWAWLGQQMQ
KRP85897    WDWLGEQIHE PASPVPAPHW APQAHPPLEL PVTPATPSEG AWAWLGQQMQ
BAL13100    WNWLGEQIQG PASPEPAPHW GAQAHPPLEL PATPATPSQG AWAWLGQQMQ
BAC47263    WNWLGEQIQG PASPEPAPHW GAQAHPPLEL PATPATPSQG AWAWLGQQMQ
APO50625    .......... .......... .......... .......... ..........
KGT81182    .......... .......... .......... .......... ..........

            551                                                  600
    InnB    EPASPSSVRP RSSNIYSGLD PLVDLDPSTP HDLHDDARSA PAPEFRRAPS
SCB50985    EPASPSSVRP RSSNIYGGLD SFVDLDPPTP HDLRDDARSA PAPEFAVTSW
KRP85897    EPGSTSSVRP RSSNIYAGLD SFVDLDPTTP HDLRDDARSA PAPEFAAASW
BAL13100    DSASPSCVRP RSSNIYGGLD SFVDLDPPHT PRLA...... ..........
BAC47263    DSASPSCVRP RSSNIYGGLD SFVDLDPPHT PRLA...... ..........
APO50625    .......... .......... .......... .......... ..........
KGT81182    .......... .......... .......... .......... ..........
```

```
           601                                               650
     InnB  FAGPSGGAQE LRDIGAVVGS DWRHGSQAAS DVLVDVLGNI NLLPNQFGPS
 SCB50985  FAGPPGPAPE LRDIGAIVGA DWRHGSQAAS DVLIDVLSNI NLLPNQFGPS
 KRP85897  FAGPPGAAPE LRDIGAIVGA NWRHGSQAAP DVLIDVLGNI DLLPNQFGPS
 BAL13100  .......... .......... .......... .......... ..........
 BAC47263  .......... .......... .......... .......... ..........
 APO50625  .......... .......... .......... .......... ..........
 KGT81182  .......... .......... .......... .......... ..........

           651                                               700
     InnB  QFMINGERYS ATFGPGGRTD VRLIHHPRPS YLDEAGPSQP LHHPPQIVQA
 SCB50985  QFAINGEHYS ATVGPEGPMD VRLIHHARAG GINEAGPSQP LYRPPQIVRA
 KRP85897  QFAINGERYS ATLGPGGRRD IRLVHHPRAG RMNEAGPSQP LHRPAPIVTA
 BAL13100  .......... .......... .......... .......... ..........
 BAC47263  .......... .......... .......... .......... ..........
 APO50625  .......... .......... .......... .......... ..........
 KGT81182  .......... .......... .......... .......... ..........

           701                                               750
     InnB  EQPREGLRDL GYLIRGGWEH RERFLPPYLV RVLQGERIMP QAGRPTYFQI
 SCB50985  ARPDEGTVDL GYLIRGGWEH RERFLPDYLV RVLEGQRIMP EVGRPTYFDI
 KRP85897  RQTGEGAVDL GYLIRGGWEH RERFLPPFLV RVLEGERIMP QPGRPTYFEI
 BAL13100  .......... .......... .......... .......... ..........
 BAC47263  .......... .......... .......... .......... ..........
 APO50625  .......... .......... .......... .......... ..........
 KGT81182  .......... .......... .......... .......... ..........

           751                       775
     InnB  RGVPYRGELD ESEGRQRVRI YPERG
 SCB50985  RGVPYRGELV ESEGRRRVRA YPERG
 KRP85897  RGMPYRGELV ESEGRQRVRV YPEPR
 BAL13100  .......... .......... .....
 BAC47263  .......... .......... .....
 APO50625  .......... .......... .....
 KGT81182  .......... .......... .....
```

Supplementary Figure S1. Alignment of InnB and its homologs in several rhizobial strains, including *B. yuanmingense* CCBAU10071 (SCB50985, 72% identity), *B. yuanmingense* BR3267 (KRP85897, 73% identity), *B. japonicum* USDA6 (BAL13100, 71% identity), *B. diazoefficiens* USDA110 (BAC47263, 70% identity), *B. diazoefficiens* USDA122 (APO50625, 70% identity) and *B. japonicum* Is-34 (KGT81182, 70% identity). Highly conserved amino acid positions are highlighted in black. The results highlighted by grey indicate the high-conserved amino acids among *B. yuanmingense* strains BR3267 and CCBAU10071.

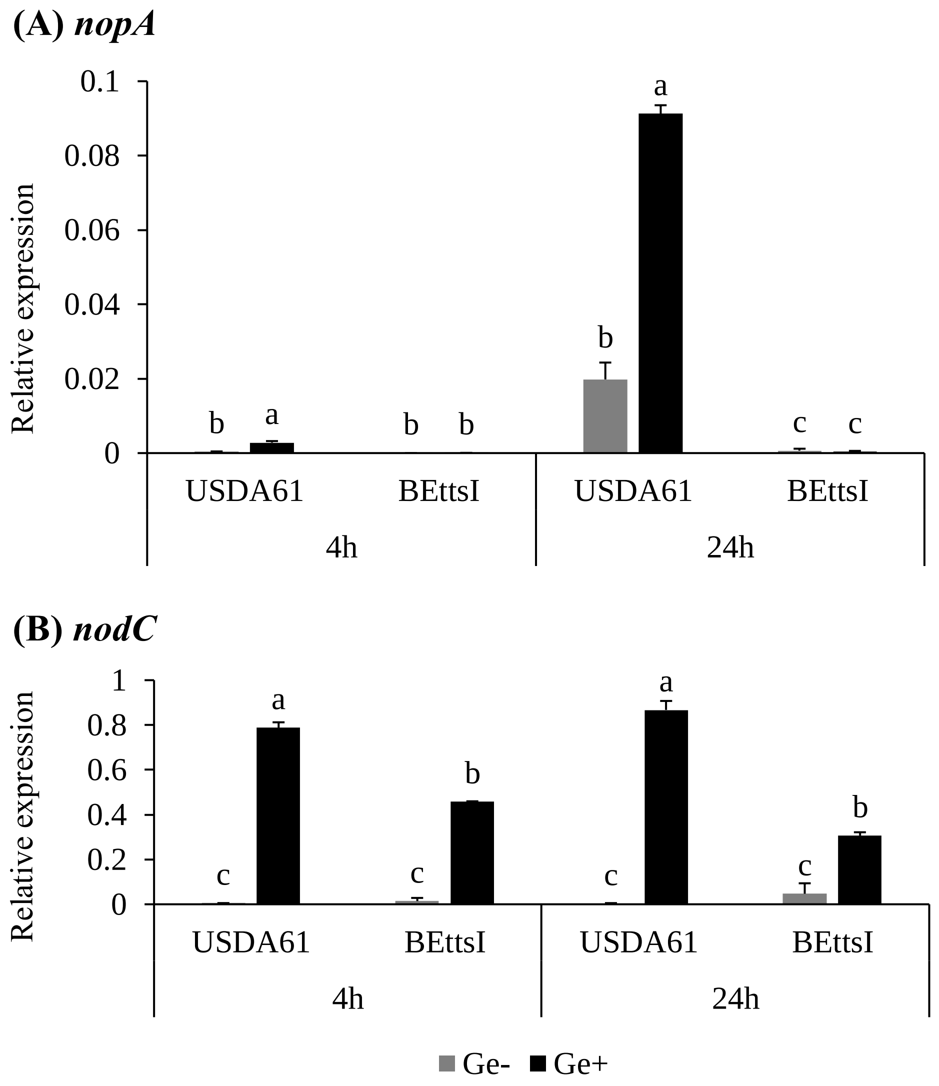

Supplementary Figure S2. Expression of *nopA* and *nodC* in wild-type and mutant *Bradyrhizobium elkanii* strains. Real-time RT-PCR was performed using total RNAs isolated from USDA61 and the *ttsI*-deficient mutant BEttsI grown in the absence (Ge-) or presence (Ge+) of the inducer flavonoid genistein (10 µM) after 4 and 24 h. The expression level of each gene was normalized relative to the *atpD* gene (ATP synthase) using the ΔΔCt method. The values are means of triplicate measurements, and the error bars indicate standard deviations. Statistical analysis by Fisher's method was performed to compare the relative expression levels of *innB* in USDA61 and BEttsI in the absence/presence of genistein after either 4 or 24 h. Means followed by different letters at the same time point are significantly different at the 5% level.

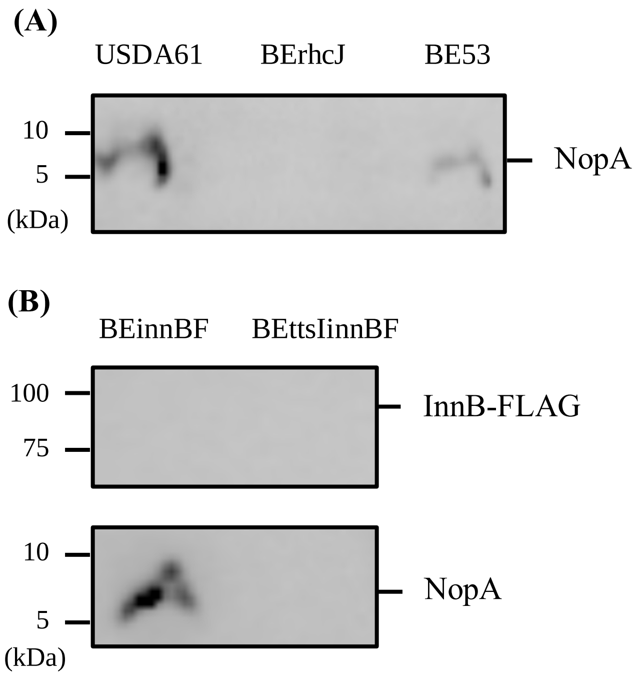

Supplementary Figure S3. Immunodetection of NopA and InnB in extracellular proteins from rhizobial cultures in presence of genistein. Proteins were separated by 4-20% and 4-15% SDS-PAGE for (A) and (B), respectively. Size-marker molecular masses (kDa) are shown on the left. Arrowheads indicate protein bands that were visible in cultures of USDA61 and BE53 but not BErhcJ.

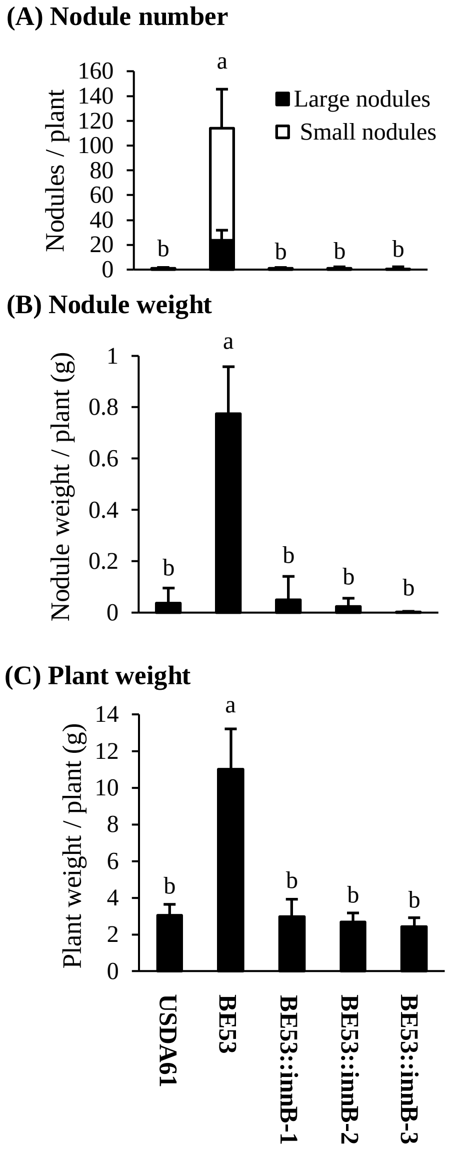

Supplementary Figure S4. Symbiotic properties of *V. radiata* cv. KPS1 inoculated with the rhizobial strains USDA61, BE53 and the *innB*-complemented mutants derived from BE53, designated BE53::innB-1, -2, and -3. (A) Nodule number, (B) nodule weight and (C) plant weight were measured at 40 dpi. At least 3 different *innB*-complemented mutants of BE53 were inoculated with KPS1 in order to confirm the symbiotic phenotypes. Open bars and closed bars in (A) show the numbers of small nodules (<2 mm) and large nodules (≥2 mm), respectively. Data showed are the mean of 6-8 plants and the error bars indicate standard deviations. Means followed by different letters are significantly different at the 5% level.

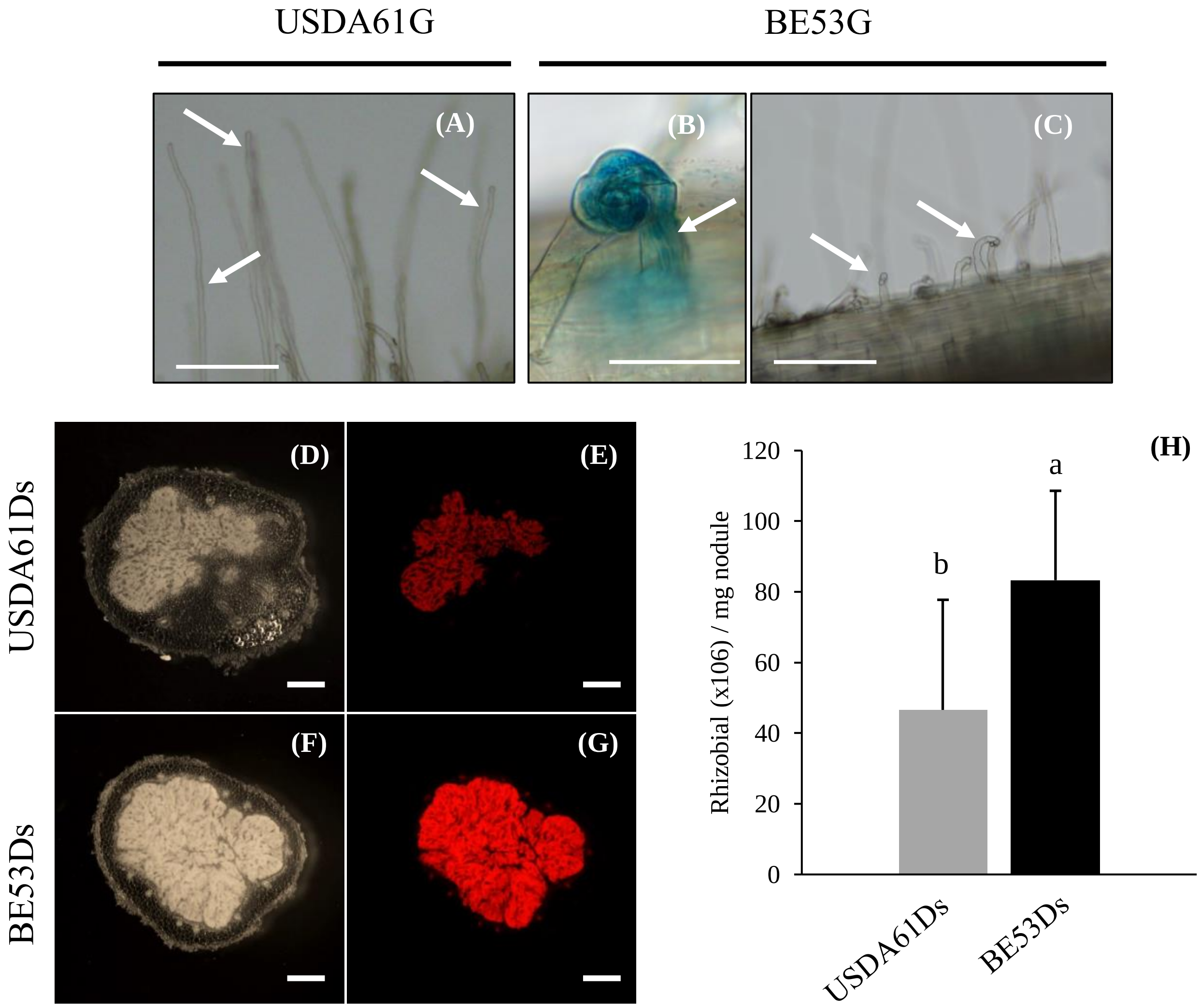

Supplementary Figure S5. (A–C) Infection threads and root hair curling of *Vigna radiata* cv. KPS1 inoculated with *Bradyrhizobium elkanii* USDA61G and BE53G at 8 dpi. Scale bars: (A) and (C), 200 µm, (B), 50 µm. (D–G) Thin sections of KPS1 nodules infected by DsRed-tagged strains of USDA61 (USDA61Ds) and BE53 (BE53Ds). (H) Numbers of reproductive rhizobia recovered from nodules. The KPS1 nodules harvested at 30 dpi were used to photograph and count the number of rhizobial cells after surface sterilization. Data shown are the mean of seven nodules selected from seven different plants, and the error bars indicate standard deviations. Means followed by different letters are significantly different at the 5% level. Scale bars: 500 µm.

2.2 Supplementary Tables

Supplementary Table S1. Bacterial strains and plasmids used in this study.

Strain or plasmid	Characteristics[a]	Reference
**Bacterial strains**		
B. elkanii		
USDA61	Wild-type strain, Pol^r	USDA[b]
BErhcJ	USDA61 derivative harboring insertion in *rhcJ* encoding a membrane protein of the type III secretion apparatus, defective in type III protein secretion, Pol^r, Km^r, Tc^r	(Okazaki et al., 2009)
BEttsI	USDA61 derivative with a deletion/insertion in *ttsI*, a positive regulator of the *tts* system, Pol^r, Sm^r, Tc^r	(Okazaki et al., 2013)
BE53	USDA61 derivative carrying a Tn*5* insertion in the *innB* gene, Pol^r, Km^r	(Nguyen et al., 2017)
USDA61Ds	USDA61 derivative containing a pBjGroEL4::DsRed2 plasmid insertion, Pol^r, Km^r, Sm^r/Sp^r	This study
USDA61G	USDA61 derivative containing a pCAM120 plasmid insertion, Pol^r, Km^r, Sm^r/Sp^r	This study
BE53Ds	BE53 derivative containing a pBjGroEL4::DsRed2 plasmid insertion, Pol^r, Km^r, Sm^r/Sp^r	This study
BE53G	BE53 derivative containing a pCAM120 plasmid insertion, Pol^r, Km^r, Sm^r/Sp^r	This study
BE53::innB 1, 2, 3	BE53 derivatives complemented with plasmid pBjGroEL4::pro*innB*, Sm^r, Pol^r, Km^r	This study
BEB759F	USDA61 derivative containing the *innB*-3xFLAG fusion integrated in the chromosome, Pol^r, Km^r	This study
BEttsIB759F	BEttsI derivative containing the *innB*-3xFLAG fusion integrated in the chromosome, Sm^r, Pol^r, Km^r	This study
BEB759C	USDA61 derivative containing the *innB-cya* fusion integrated in the chromosome, Pol^r, Sm^r, Tc^r	This study
BErhcJB759C	BErhcJ derivative containing the *innB-cya* fusion integrated in the chromosome, Pol^r, Sm^r, Tc^r, Km^r	This study
E. coli		
HB101	*recA*, *hsdR*, *hsdM*, *pro*, Sm^r	Invitrogen
S17-1	*thi pro hsd*R$^-$ *hsd*M$^+$ *recA* RP4::2-Tc::Mu-Km::Tn*7*(Tp^r/Sm^r)	(Simon et al., 1983)
**Plasmids**		
pRK2013	Helper plasmid, ColE1 replicon carrying RK2 transfer genes; Km^r, tra	(Figurski and Helinski, 1979)
pCAM120	mTn*5*SS*gusA20* (P*aph-gusA-trpA* ter translational fusion) in pUT/mini-Tn*5*; Sm^r/Sp^r, Ap^r	(Wilson et al., 1995)
pBjGroEL4::DsRed2	*dsRed* transposon delivery vector, Sm^r/Sp^r	(Hayashi et al., 2014)
pBjGroEL4::proinnB	pBjGroEL4 carrying a ~2.6-kb DNA fragment containing *innB*, its upstream *tts* box and promotor, Ap^r	This study
pK18mob	Cloning vector, pMB1 oriV, *oriT*, Km^r	(Schäfer et al., 1994)
pK18mob3xFLAG	pK18mob containing the 3x FLAG, used for the generation of translational fusions with the 3x FLAG, Km^r	This study
pInnB7593xFLAG	pK18mob3xFLAG containing a ~0.5-kb DNA fragment from the stop codon of *innB* thereby generating an *innB*-3xFLAG fusion at the 3'-end, Km^r	This study
pSLC5	pSUPPOL2SCA containing the *cya* reporter gene, used for the generation of translational fusions with *cya*, Tc^r	(Wenzel et al., 2010)
pSLC5Sm	pSLC5 containing 1,536 bp of Sm resistance gene (*aadA*) and its promotor in *Dra*I site, Sm^r, Tc^r	This study
pInnB759Cya	pSLC5Sm containing a ~0.5-kb DNA fragment from the stop codon of *innB* coding region thereby generating an *innB-cya* fusion at the 3'-end, Sm^r, Tc^r	This study

[a]Pol^r, polymyxin resistant; Km^r, kanamycin resistant; Sm^r, streptomycin resistant; Sp^r, spectinomycin resistant; Tc^r, tetracycline resistant; Tp^r, trimethoprim resistant; Ap^r, ampicillin resistant.
[b]United States Department of Agriculture, Beltsville, MD.

References

Figurski, D. H., and Helinski, D. R. (1979). Replication of an origin-containing derivative of plasmid RK2 dependent on a plasmid function provided in trans. *Proc. Natl. Acad. Sci. U. S. A.* 76, 1648–52.

Hayashi, M., Shiro, S., Kanamori, H., Mori-Hosokawa, S., Sasaki-Yamagata, H., Sayama, T., et al. (2014). A Thaumatin-Like Protein, Rj4, Controls Nodule Symbiotic Specificity in Soybean. *Plant Cell Physiol.* 55, 1679–1689. doi:10.1093/pcp/pcu099.

Nguyen, H., Miwa, H., Kaneko, T., Sato, S., and Okazaki, S. (2017). Identification of *Bradyrhizobium elkanii* genes involved in incompatibility with *Vigna radiata*. *Genes (Basel).* 8, 374. doi:10.3390/genes8120374.

Okazaki, S., Kaneko, T., Sato, S., and Saeki, K. (2013). Hijacking of leguminous nodulation signaling by the rhizobial type III secretion system. *Proc. Natl. Acad. Sci. U. S. A.* 110, 17131–6. doi:10.1073/pnas.1302360110.

Okazaki, S., Zehner, S., Hempel, J., Lang, K., and Göttfert, M. (2009). Genetic organization and functional analysis of the type III secretion system of *Bradyrhizobium elkanii*. *FEMS Microbiol. Lett.* 295, 88–95. doi:10.1111/j.1574-6968.2009.01593.x.

Schäfer, A., Tauch, A., Jäger, W., Kalinowski, J., Thierbach, G., and Pühler, A. (1994). Small mobilizable multi-purpose cloning vectors derived from the *Escherichia coli* plasmids pK18 and pK19: selection of defined deletions in the chromosome of *Corynebacterium glutamicum*. *Gene* 145, 69–73.

Simon, R., Priefer, U., and Pühler, A. (1983). A Broad Host Range Mobilization System for *In Vivo* Genetic Engineering: Transposon Mutagenesis in Gram Negative Bacteria. *Bio/Technology* 1, 784–791. doi:10.1038/nbt1183-784.

Wenzel, M., Friedrich, L., Göttfert, M., and Zehner, S. (2010). The type III–secreted protein NopE1 affects symbiosis and exhibits a calcium-dependent autocleavage activity. *Mol. Plant-Microbe Interact.* 23, 124–129. doi:10.1094/MPMI-23-1-0124.

Wilson, K. J., Sessitsch, A., Corbo, J. C., Giller, K. E., Akkermans, A. D. L., and Jefferson, R. A. (1995). β-Glucuronidase (GUS) transposons for ecological and genetic studies of rhizobia and other Gram-negative bacteria. *Microbiology* 141, 1691–1705. doi:10.1099/13500872-141-7-1691.

Supplementary Table S2. DNA oligonucleotide primers used in this study.

Oligonucleotides	Sequences	Source	Usage
3FLAPstI	5'-GGATTACAAGGATGACGACGATAAGGACTATAAGGACGATG ATGACAAGGACTACAAAGATGATGACGATAAATAGGCATG-3'	This study	Construction of pK18mob3xFLAG
3FLASphI	5'-CCTATTTATCGTCATCATCTTTGTAGTCCTTGTCATCATCGTC CTTATAGTCCTTATCGTCGTCATCCTTGTAATCCTGCA-3'	This study	Construction of pK18mob3xFLAG
aadAfor	5'-TGATTTGCTGGTTACGGTGA-3'	This study	Amplification of Sm resistance gene
aadArev	5'-TACTGCGCTGTACCAAATGC-3'	This study	
BeatpD-F	5'-GGTCGTCGATCTTCTTGCTC-3'	This study	RT-PCR assays
BeatpD-R	5'-CGGCGAACACGGAGTAAC-3'	This study	
BenodC-F	5'-CGTGGAAAGCCAAGTGATTT-3'	This study	RT-PCR assays
BenodC-R	5'-TTGCTGTCGCAGATATGGTC-3'	This study	
BenopA_F	5'-CGCAGCTGGTACTGCTACTG-3'	This study	RT-PCR assays
BenopA_R	5'-GAGACGACGCGAAGTTCTACA-3'	This study	
BeinnBRT-F	5'-TTTGCGCGATGATCTATTCA-3'	This study	RT-PCR assays
BeinnBRT-R	5'-GGACTTGCTACTCCCTCCAA-3'	This study	
BeinnBSacI-InfuF	5'-GGGAACAAAAGCTGGAGCTCAGGAACGCTTACGGGCTGTCTTCTC-3'	This study	Complementation of *innB*
BeinnBKpnI-InfuR	5'-GCTAGGGCGAATTGGGTACCTCAGCCACGTTCAGGATAGATGCGA-3'	This study	
PsuppolF	5'-ATAAACCAGCCAGCCGGAA-3'	This study	Confirmation of integration of *cya* fusions
PsuppolR	5'-TTCTGACAACGATCGGAGGA-3'	This study	
M13 forward (-20)	5'-GTAAAACGACGGCCAGT-3'	This study	Confirmation of integration of 3xFLAG fusions
M13Rev0	5'-TTCCGGCTCGTATGTTGTGT-3'	This study	
Be53-F	5'-ACCTTGCCATCACGATTAGC-3'	This study	Confirmation of integration of *cya* and 3xFLAG fusions
Be53-R	5'-GCCTCGGTCATCAAACATCT-3'	This study	
innB-PstI-fullF	5'-CCTTGTAATCCTGCAGGCCACGTTCAGGATAGATGC-3'	This study	Construction of the *innB*-3xFLAG fusion
innB-BamHI-fullR	5'-CGGTACCCGGGGATCCCAGGAACTGCGGGATATTG-3'	This study	
aadA-DraI-F	5'-ACTTTAGATTGATTTAAATCCAAACGAGAGTCTAATA-3'	This study	Cloning Sm resistance gene
aadA-DraI-R	5'-CACCTAGATCCTTTTAAATTGACCTGATAGTTTGGCTGTGAG-3'	This study	
innB-EcoRI-fullF	5'-GATGCCCGGGGAATTCCAGGAACTGCGGGATATTG-3'	This study	Construction of the *innB-cya* fusion
innB-XbaI-fullR	5'-TTGCTGCATATCTAGACAGCCACGTTCAGGATAGATGC-3'	This study	
